# A Multicentric and Retrospective Clinical Study: 2 Year Follow-up Results for Breast Surgery With Perle Smooth Opaque Silicone Breast Implants

**DOI:** 10.1093/asjof/ojae029

**Published:** 2024-04-24

**Authors:** Jean-Luc Jauffret

## Abstract

**Background:**

For breast surgery, there are a number of implants available that offer different options, such as shape, surface characteristics, gel fillers, and size/profile to meet the diverse needs of the patient population. Each implant option has its own advantages and disadvantages, and the individual patient’s needs should be taken into account when making choices.

**Objectives:**

To conduct an assessment of the safety, performance, and satisfaction rates through a 2-year follow-up study for the Perle mammary implant (Nagor Ltd, Glasgow, Scotland, United Kingdom).

**Methods:**

A retrospective, observational, multicenter, noncomparative study was conducted from March 2023 to June 2023. The collection of data took place in 5 centers across France and Italy. Patients included in the study received the evaluated device between October 2020 and June 2021 for cosmetic and medical indications.

**Results:**

Of the 97 patients included in the study, only 9 patients reported complications during the 2 years following the surgery. The overall complication rate was 9.28%. All patients and surgeons were satisfied or very satisfied with the surgery and the implant.

**Conclusions:**

This patient cohort showed a rate of complications consistent with the range of other smooth implants after 2 years of follow-up and a high rate of both patient and surgeon satisfaction.

**Level of Evidence: 4:**

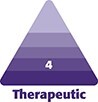

Breast implants have emerged as a validated and often preferred option for breast reconstruction and aesthetic enhancement. The growing demand for optimal aesthetic results, coupled with constant advances in materials and surgical techniques, has led to a rapid expansion in this field. In 2022, the International Society of Aesthetic Plastic Surgery, reported that 2,174,616 breast augmentation surgeries were performed worldwide, an increase of 29% from 2021.^1^

Since their introduction several decades ago, these medical devices have undergone significant technological evolution in terms of shells, surface characteristics, shape, and filling gel. Silicone has been used as a filler since the invention of the first implants in the 1960s.^2^ However, it has evolved over the years to become a more pure, reliable, and durable filler, preventing leakage in the event of implant rupture, and giving a more natural postimplant impression. Currently, the fifth- and sixth-generation gels are used in silicone-filled implants. Products on the market today are designed with improved materials, quality control, and stricter criteria in terms of shell thickness and gel cohesion. The breast implant shape has also evolved, with both round and anatomical options now available. The concept of texturing the surface of implants began in the 1970s with the aim of mitigating complications, such as capsular contracture or malposition.^2,3^

Recently, some generations of breast implants have been affected by concerns over the appearance of a hematological cancer called breast implant-associated anaplastic large cell lymphoma (BIA-ALCL).^4^ In 2017, the SCHEER Committee (Scientific Committee on Health, Environmental, and Emerging Risks) conducted a literature search to gather evidence about the possible association between breast implants and ALCL.^5^ In April 2021, the committee concluded that although BIA-ALCL is uncommon (ie, it has a very low incidence) and multifactorial, there was a moderate weight of evidence for a causal relationship with textured implants.^6^ SCHEER also concluded that it is important that this risk should be weighed against the benefits for each implant type.^6^

It is known that each type of implant has some advantages that will benefit some patients’ needs. However, they also have some associated complications, which need to be weighed against the benefits when making an implant choice. Traditional smooth implants have been historically associated with more capsular contracture, especially when the implant is inserted in the subglandular plane,^7^ more rotation, malposition, bottoming out, and lateralization.^8,9^ Textured implants have been reported as being more associated with late seromas, double capsules, and BIA-ALCL.^7^

As surgeons, we always make choices based on what is best for our patients, and we select the appropriate implant shape and surface according to patient characteristics and the desired outcome. There is also a need to consider the safety of the implant to ensure the lowest complication rates for our patients.

As breast implants continue to play a central role in the field of plastic and reconstructive surgery, products on the market have evolved to adapt to emerging risks, and alternatives have been developed with different types of textured and smooth surfaces. It is imperative that the safety and performance of these new devices are critically evaluated to guide practitioners and patients toward the best informed decisions. This review aims to provide early follow-up information on this new CE marked smooth opaque^10^ round-shaped breast implant, designed to meet these challenges.

## METHODS

This study was designed to be retrospective, observational, multicentric, and noncomparative and was conducted from March 2023 to June 2023. The occurrence of early complications as well as patient and surgeon satisfaction was collected at a follow-up period from 21 to 32 months. It was a manufacturer-led study performed across 5 sites in France and Italy. As per local regulations for retrospective studies, no ethics committee was involved. However, the study was compliant with the Declaration of Helsinki.

The inclusion criteria were that the patients should be females aged between 18 and 65 years. Patients were included if they had been implanted with the evaluated device for medical or cosmetic purpose. The patients were required to have gone through their surgery on average 2 years before this investigative study (21-32 months).

Patients were excluded from the study if the surgery did not comply with the indications, conditions, or exclusion criteria advised in the Instruction for Use.

The data were collected as per the General Data Protection Regulation from the surgical files of patients who met the study criteria. Clinical information was collected from charts as per local hospital protocol, and anonymized data were transferred to case report forms whereby patients were identified by their study number.

The evaluated device was a newly CE marked, available since 2020 in the European market. Perle is a smooth, round mammary implant (Nagor Ltd, Glasgow, Scotland, United Kingdom). The shell of the implant has a surface roughness of 5 microns, which is classified as a smooth implant according to ISO 14607 (2018).

In this study, descriptive statistical analyses were employed. Numbers were presented as means with ranges as appropriate, or as percentages.

## RESULTS

From March 2023 to June 2023, 97 patients met the inclusion and exclusion criteria and were included in the analysis. On average, the follow-up time within this group was 26 months (range, 21-32 months). All patients had bilateral surgery but 3 of the patients received the evaluated breast implant only on one side. The data were collected from France (*n* = 41 patients) and Italy (*n* = 56 patients).

Most of the patients were aged between 30 and 39 years (55 patients, 56.7%). The mean age of the patients was 33 years (range, 17-60 years).

The average height and weight were 165 cm (range, 157-178 cm) and 56 kg (range, 43-85 kg), respectively.

Patients were indicated for the surgery for medical or cosmetic reasons and for revision surgery, as presented in Table 1.

**Table 1.** Indications for Breast Surgery

Indications	Number	Percentage
Augmentation/cosmetic purpose only	33	34
Medical reason	49	50.5
Revision surgery	15	15.5

Of the 97 patients included, 85 (88%) received implants only for breast surgery. For the 12 other patients (12%), surgeons reported additional procedure, such as fat grating or total capsulectomy. Patients underwent local anesthesia with sedation (*n* = 55, 57%), or general anesthesia (*n* = 42, 43%).

A summary of the perioperative information is presented in Table 2.

**Table 2.** Surgery Information

	No. of patients (%)
Incision size, cm	
0-3	46 (47.4)
3-6	46 (47.4)
6-9	5 (5.2)
Incision site	
Periareolar	57 (58.8)
Inframammary fold	35 (36.1)
T-inverse McKissock technique	5 (5.1)
Breast implant placement	
Submuscular	40 (41.2)
Dual plane	39 (40.2)
Subglandular	19 (19.6)

The intraoperative care used during surgery includes pocket irrigation (*n* = 57, 58.8%), antibiotics (*n* = 95, 98%), drains (*n* = 71, 73.3%), Betadine (Purdue Products L.P.; *n* = 40, 41.2%), HydroCone (GCA, Ireland) to ease implant insertion and minimal touch technique (*n* = 20, 20.6%), and the use of steroids (*n* = 1, 1%).

The model and size of implants were recorded for each patient included in the study. A total of 191 implants were used in 97 patients.

**Figure 1.**
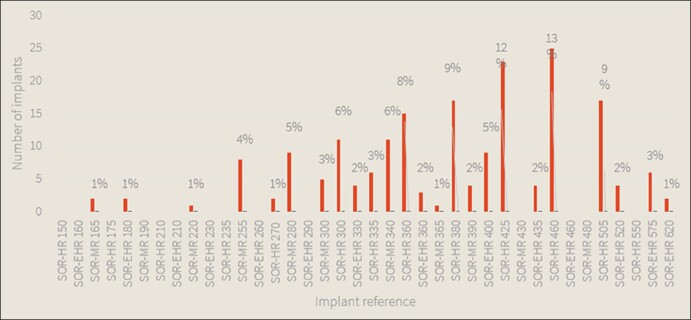
Implant size distribution. This figure presents the different implant size and projection used in this study. Perle is available in 3 profiles: medium profile (SOR-MR), high profile (SOR-HR), and extra-high profile (SOR-HER).

The implant size distribution is spread across 3 profiles: medium profile (SOR-MR), high profile (SOR-HR), and extra-high profile (SOR-HER). The most common implant size used overall was the high profile 460 cc (*n* = 25 implants, 13%). The size distribution is presented in Figure 1.

Out of the 97 patients, complications were reported by 9 individuals 2 years post-surgery, resulting in a complication rate of 9.28%. These 9 patients experienced 12 complications. One patient experienced 3 complications: implant malposition on the left and 2 explantations because she did not tolerate the implants. She was nevertheless satisfied because she already had a history of implant intolerance. One other patient experienced both implant malposition and asymmetry that subsequently resolved. The 7 other patients experienced only one complication. A breakdown of all the complications is summarized in Table 3. Two patients had several complications.

**Table 3.** Breakdown of Complications

Complication	Occurrence
Explantation	2 (2%)
Asymmetry	2 (2%)
Implant displacement/rotation/extrusion/flipping	2 (2%)
Pain	1 (1%)
Capsular contracture	4 (4.1%)
Seroma	1 (1%)

Patients and surgeons’ satisfaction was collected through a nonstandardized questionnaire. Patients and surgeons were asked whether they were satisfied with the outcome of the surgery and had the possibility in answering positively or negatively. The satisfaction was then based on no specific criteria but a general appreciation of the overall outcome. Despite the limited number of proposals, satisfaction was collected for all 97 patients, and they reported to be satisfied (*n* = 97, 100%) with the outcome of the surgery. One hundred percent of surgeons also reported to be satisfied with the outcome of the surgery.

## DISCUSSION

Malposition and capsular contracture are the main expected early complications and the most common reasons for implant revision.^10-12^ Malposition includes lateral displacement, rotation, bottoming out, or implant flipping. This complication may require surgical intervention or manual manipulation to reposition the prosthesis correctly. Malposition has been found to be more severe with smooth implants compared with textured implants.^12^ Textured breast implants were developed to decrease the risk of capsular contracture and malposition but some recent concerns regarding BIA-ALCL linked to textured devices have led to new innovations in breast implant design. The risk of capsular contracture has been reported to be increased for smooth surface devices.^10^ Other factors than the smooth surface of the implant may also increase the risk of capsular contracture, severe malposition, and the risk of secondary procedures. Some studies reported that periareolar or axillary incision may increase these risks compared with inframammary incision site.^11^ Another study reported no significant difference in the incidence of capsular contracture between smooth and textured implants when using the inframammary approach.^13^

Surgery techniques using smooth implants compared with textured implants should be more precise in terms of dissection of the pocket to yield good results and minimize the risk of malpositionning. The 2% rate of the patients experiencing malposition in this study is below the 6% rate for other traditional smooth devices and aligned with the 1.4% rate for textured devices reported in the literature.^11^ In the literature, the rate of capsular contracture for smooth implants with a short-term follow-up has been reported to be between 0% and 20.6%.^14-17^ The rate of capsular contracture rate found in this study (4.1%) is consistent with the range found in the literature for smooth implants. Long-term follow-up with a larger patient cohort would be of interest to provide further evidence regarding the complication rate observed in this study.

Another paper has recently been published on this implant with a similar follow-up length, reporting 5.6% overall complications in a larger cohort (374 patients), which is lower than reported in this study.^14^ The authors reported 1.6% implant displacement, in accordance with this study and 0% of capsular contracture (4.1% in this study). This difference may be explained by the shorter follow-up time of 18 months on average (6-30 months).^14^

This new smooth implant has a higher surface roughness compared with traditional smooth implants, which could potentially decrease the likelihood of malposition and capsular contracture and alleviate concerns associated with BIA-ALCL. More clinical data are required to confirm this presumption.

The limitations of this study include the retrospective design, which does not enable the patient to fill out a well-being questionnaire, the small cohort, and the absence of a comparator group. This work does not aim to conclude alone on the safety benefit profile of this implant. Long-term experience with a larger cohort and standardized satisfaction questionnaires are needed to confirm the long-term maintenance of complication rates observed in this study, as capsular contracture rate, malposition, or patient satisfaction can change over decades, but preliminary data show a good balance between safety, performance, and satisfaction rates.

## CONCLUSIONS

This multicentric study showed that this implant demonstrated promise with favorable outcomes and consistent complication rates. However, further validation is required through larger cohorts and extended follow-up periods. The observed safety and performance over a 2-year period suggest that this type of implant addresses current challenges and meets the needs of both patients and surgeons, as indicated by satisfaction results. The implant has a good risk/benefit balance with complication rates comprised between those expected for traditional smooth implants and textured implants.
